# BIRCH: A user-oriented, locally-customizable, bioinformatics system

**DOI:** 10.1186/1471-2105-8-54

**Published:** 2007-02-09

**Authors:** Brian Fristensky

**Affiliations:** 1 Department of Plant Science, University of Manitoba, Winnipeg, Manitoba, R3T 2N2, CANADA

## Abstract

**Background:**

Molecular biologists need sophisticated analytical tools which often demand extensive computational resources. While finding, installing, and using these tools can be challenging, pipelining data from one program to the next is particularly awkward, especially when using web-based programs. At the same time, system administrators tasked with maintaining these tools do not always appreciate the needs of research biologists.

**Results:**

BIRCH (Biological Research Computing Hierarchy) is an organizational framework for delivering bioinformatics resources to a user group, scaling from a single lab to a large institution. The BIRCH core distribution includes many popular bioinformatics programs, unified within the GDE (Genetic Data Environment) graphic interface. Of equal importance, BIRCH provides the system administrator with tools that simplify the job of managing a multiuser bioinformatics system across different platforms and operating systems. These include tools for integrating locally-installed programs and databases into BIRCH, and for customizing the local BIRCH system to meet the needs of the user base. BIRCH can also act as a front end to provide a unified view of already-existing collections of bioinformatics software.

Documentation for the BIRCH and locally-added programs is merged in a hierarchical set of web pages. In addition to manual pages for individual programs, BIRCH tutorials employ step by step examples, with screen shots and sample files, to illustrate both the important theoretical and practical considerations behind complex analytical tasks.

**Conclusion:**

BIRCH provides a versatile organizational framework for managing software and databases, and making these accessible to a user base. Because of its network-centric design, BIRCH makes it possible for any user to do any task from anywhere.

## Background

The diversity of computational tools needed in genomics presents many challenges [1]. The most sophisticated algorithms and methods might as well not exist if the programs implementing them are not accessible to the biologist. Biologists need to understand the tasks with which they are faced, find software tools that will help them with these tasks, and be able to evaluate the validity and significance of the results.

Many packages provide a large set of programs and functions, and to different degrees, try to promote usability through graphic interfaces, hierarchical desktop menus, and organization of documentation files. To simplify comparison with BIRCH, a few representative packages will be cited as examples. Packages such as EMBOSS [2] and Staden [3] are available for many platforms. There are numerous other packages specific to Linux. BioRPMS is a collection of packages that can be installed on an existing Linux system [4], while NEBC Bio-Linux [5] is a complete Linux distribution with bioinformatics applications pre-installed. In some cases, graphic interfaces are available to tie together sets of applications. Two examples include JEMBOSS [6], a Java front end for EMBOSS, and the Kaptain extensions to EMBOSS [7], which utilize Kaptain, a system for generating graphic interfaces for commandline programs using grammar scripts [8]. Similarly, web-based interfaces to over 200 applications have been generated using Pise, which creates HTML interfaces from XML definitions of program parameters [9]. The Taverna workbench takes a different approach. Taverna is a Java application in which complex data workflows can be created by linking together icons representing web services available at both local and remote sites [10].

Bioinformatics packages such as those described above can be made available to users throughout a lab, department, or campus on network-centric Unix/Linux systems. Network-centric systems allow users to login from any computer and be presented with the same desktop, programs, and filesystems [11]. This avoids some of the problems associated with individual PCs, because users don't have to be worried about which software is installed on which PC. While installing programs and databases can be a major effort, transforming them from a mere collection into an integrated system is far more difficult [12,13].

The BIRCH system consists of a core of commonly-used programs for most typical bioinformatics tasks, set within a portable framework that allows for seamless integration of locally-installed programs so that each BIRCH site can be tailored to the needs of the local user-community. GDE [14] as updated by Eric Linton [15] provides a powerful graphic interface unifying both core BIRCH programs and locally-installed software. The BIRCH documentation system provides a merged view of both core and locally-installed documentation. BIRCH also contains numerous tools that make it easier for the system administrator to manage, update and customize the system for the local user base.

## Implementation

### I. The user' perspecitve

#### A seamless view of the software

The BIRCH core distribution comes with a wide range of commonly-used software packages pre-configured and ready to run. These include NCBI network BLAST, Cn3D, and Sequin [16], FASTA [17], PHYLIP [18], TCOFFEE [19], and Taverna [10]. All programs can be run from the command line, and most can also be launched from GDE.

GDE can be thought of as a program that runs other programs. The flexibility of GDE makes it possible to have GDE interfaces specialized for different types of data. In the current implementation of BIRCH, there are four GDE interfaces:

• GDE – sequence data

• dGDE – list data (eg. ACCESSION, GI, TAXID numbers)

• mGDE – molecular marker data (eg. AFLP)

• tGDE – phylogenetic tree data

**Figure 1:**
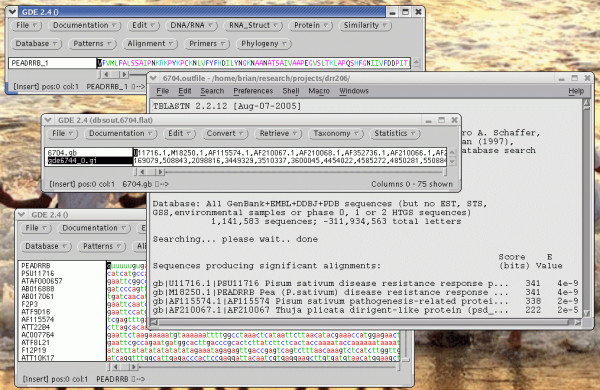
**Creation of a dataset of plant defense gene sequences**. To find genes related to pea defense protein DRR206, the DRR206 protein (PEADRRB) was selected in the GDE window (top), and TBLASTN was launched from the Database menu. The results appear in two windows (middle). The BLAST report appears in a text editor, and the accession numbers appear in a dGDE window. dGDE is a GDE implementation specialized for working with lists of identifiers. The list of accession numbers was selected in the dGDE window, and a request sent to the SeqHound data warehouse [27], retrieving the corresponding NCBI GI numbers. Next, the GI numbers were selected, and sequences were retrieved from SeqHound. The new GDE window (bottom), contains DNA sequences for all of the BLAST hits.

**Figure 2:**
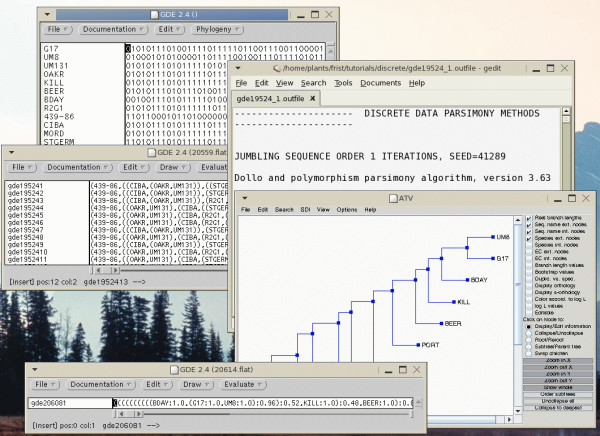
**Phylogenetic analysis of plant populations**. To evaluate population structure in the weed green foxtail, RAPD marker data from different foxtail accessions were read into an mGDE window (top), and selected. Parsimony analysis was chosen from the Phylogeny menu, and the Phylip DOLLOP program generated a set of equally parsimonious trees, which appear in a tGDE window, and in the text report (middle). All trees were selected, and CONSENSE was run. Output is a single consensus tree, which appears in a new tGDE window, as well as in the ATV tree editor (bottom). The tGDE window has additional menu options for running various other tree display programs.

The functions of these interfaces will be illustrated in two examples. GDE lends itself to complex tasks in which data is pipelined from one step to the next. Figure 1 shows a simple example of how to create a dataset of sequences for a specific gene family, beginning with a single amino acid sequence. Figure 2 shows an example of building a phylogenetic tree from molecular marker data, using mGDE and tGDE.

With interfaces such as Jemboss, Kaptain, or Pise, input and output files for a given program are selected within the menu that launches the program. A program is selected from a list, one or more datafiles are chosen, parameters are set, and the program is launched. Thus, at each step in the analysis, the user must ensure that an input file, of the type expected by the program, is available. In contrast, data need only be read into GDE at the first step. Data items (eg. sequences, trees) are displayed in the GDE window, and can be selected individually, or in any combination, prior to launching a program. Even parts of sequences can be selected with the mouse. GDE automatically converts data into the format required for each subsequent step. Finally, whereas GDE can send output to a new GDE window for further processing, Jemboss and Kaptain require that output be saved to a file before it can be used as input for the next analytical step.

BIRCH uses GDE in several ways to overcome the learning curve usually associated with trying a new program. First, all of the "overhead" tasks associated with running a program (eg. interconversion of file formats using READSEQ [20]) are automated in wrapper scripts. As well, wrappers can prevent errors by checking the validity of the parameters set by the user. Long-running programs that are CPU intensive are run in the background at lower priority, minimizing the impact on system performance. When jobs are run in the background, the user can logout and review the results at a later time when the job is complete.

**Figure 3:**
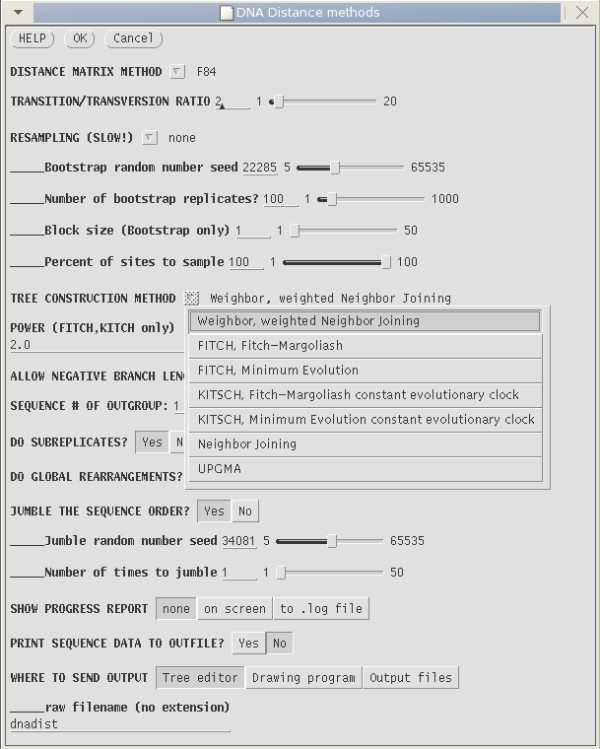
**Implementation of an analysis pipeline in a single menu**. Parameter settings for all steps in construction of a DNA distance tree are incorporated into the DNA Distance methods menu of GDE. All programs in the pipeline are from the Phylip package [18], with the exception of WEIGHBOR, which combines elements of maximum likelihood methods and distance methods [28].

Figure 3 illustrates how a complex pipeline can be implemented in a single menu, using the example of construction of a DNA sequence distance tree from a multiple alignment. From the user's point of view the entire pipeline appears to be executed as a single step, even though numerous programs are run. If bootstrap resampling is chosen, the script will first generate a bootstrapped dataset using SEQBOOT. Next, DNADIST is called to calculate the distance matrices, which are used as input for one of the phylogeny programs shown in the pulldown menu in Figure 3 (WEIGHBOR, FITCH, KITCH or NEIGHBOR, which together implement seven distinct methods). If bootstrapping was done, a consensus tree is automatically generated by CONSENSE. The output report pops up in a text editor, and trees pop up in a text editor and tree editor, or are written to files. In this way, the user is presented with a variety of choices for viewing or further analysis of the data.

#### GDE is a programmable interface

By itself, GDE is a small program that can read and write datafiles, display the data as a set of character strings, and create menus. All programs that GDE runs are completely external to GDE, and are not compiled as part of the code. (In fact, almost the only component remaining from the original GDE distribution [14] is the GDE interface itself. Virtually all menus, and most of the external programs, are new to BIRCH.) When GDE is started, it reads a set of menu items for each program. Each menu item has a template for a Unix command to be executed, along with parameters that are displayed in menus as buttons, sliders, choosers, or text. In this regard, the GDE menu syntax is analogous to the ACD command definition syntax used for describing program input, output and parameters in EMBOSS, or the XML program descriptions in Pise. In most cases, the program called by GDE is a wrapper script, which might execute a single command, or a complex series of commands needed to accomplish a task. To run a program from GDE, the user reads in a datafile (eg. sequences), selects the data to be analyzed, and then chooses a program from the menus. Next, the user sets parameters and clicks on the OK button. Parameter settings are substituted into the Unix command template, and the command is executed. Programs launched by GDE do not even need to be running on the same login host or workstation to which the user is logged in, because anything that can be run from a script can be run by GDE, including web services on remote systems. In the core BIRCH distribution, BLAST searches are run remotely at NCBI using the NCBI BLASTCL3 client.

Wrapper scripts can also enhance the presentation or content of the output. For long-running programs, scripts will add the name of the login host and time and memory resources utilized, as a guide for scaling up to larger jobs. Some programs, as written, do not include in the output potentially important information needed for interpreting the results. In such cases, BIRCH wrapper scripts will add more detailed information on parameters and input files used in each run.

#### Any user can do anything from anywhere

**Figure 4:**
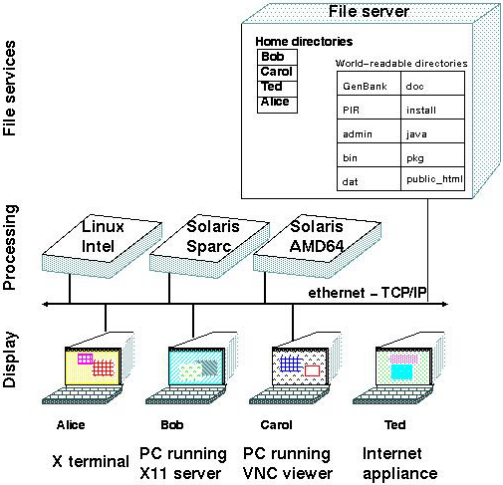
**Scalability of a BIRCH system**. On a PC, File Services, Processing and Display would all reside on the PC. Alternatively, a Unix/Linux workstation might remotely-mount filesystems, but carry out the processing and display steps. For larger multiuser systems, File Services might be remotely-mounted onto login servers, where programs actually run. Windows generated by programs would appear on the local display, which could be an X-terminal or other thin client, or a PC running X11 display software. At login, BIRCH determines the OS/hardware platform of the login host (eg. Linux-Intel, Solaris-Sparc or Solaris-AMD64) and provides the user with platform-specific binaries, libraries, paths and environment variables.

BIRCH is scaleable from a single workstation to a server cluster. Figure 4 illustrates a typical campuswide system. By choosing binaries and libraries at login, BIRCH makes it transparent to the user which platform they are actually using. Most importantly, a Unix graphic desktop can be redirected from the server to be displayed anywhere. BIRCH has been successfully tested on X-terminals, SunRay terminals, PCs running X11 clients such as XWin32 [21] and CygWin [22], Sun Secure Global Desktop [23] and various versions of VNC [24]. BIRCH has also been tested on several graphic desktops, including CDE, GNOME, Sun Java Desktop and KDE. In our laboratory, it has been possible to eliminate PCs entirely, and instead do all work, both common office tasks and specialized bioinformatics tasks, from low-cost terminals.

### II. Local customization of a birch system

No software package does everything, and each lab, department, or institution has different needs. BIRCH has numerous mechanisms for adding programs and documentation that are not part of the BIRCH core, and for customization to take advantage of the strengths of a local local Unix/Linux system, and to work around problems specific each system.

#### The $BIRCH/local directory

BIRCH is downloaded as a hierarchical directory structure which is usually installed in the $HOME directory of an account specifically used for administering BIRCH. This directory is referred to by the $BIRCH environment variable. Local customization is made possible through the $BIRCH/local directory. Analogous to /usr/local in Unix, $BIRCH/local is a part of the system that does not change when an updated version of BIRCH is installed. During an update the birchconfig install wizard automatically merges programs, documentation, and settings from $BIRCH/local into the new version of BIRCH. For example, to add a program to a BIRCH system on Linux, the binaries would be added to $BIRCH/local/bin-linux-intel, and the documentation added to $BIRCH/local/doc. At the same time, software packages already in place prior to installing BIRCH can be integrated into BIRCH simply by creating symbolic links to them from $BIRCH/local.

#### Automatic updating of GDE interfaces

$BIRCH/local contains directories for adding menus to each of the four GDE interfaces (dGDE, GDE, mGDE and tGDE). To add a program, the binary file is copied to $BIRCH/local, and then a GDE menu file is written, typically by modifying an existing menu file. A script merges the new menu into the existing menus. Subsequently, when BIRCH is updated to a new version, the menu is automatically added to GDE. For example, the core BIRCH distribution runs BLAST remotely at NCBI. When BIRCH was installed at the University of Calgary, symbolic links were made to binaries for the already existing Paracel^® ^BLAST system [25], and menus for launching Paracel BLAST were added by copying the NCBI BLAST menus to $BIRCH/local and modifying them.

#### Working in a heterogeneous computing platform

BIRCH has unique design considerations for working in a heterogeneous operating environment consisting of workstations and hosts with different operating system/hardware platforms. For example, the Unix system at the University of Manitoba is configured as shown in Figure 4. Users can log into machines running either Linux, Solaris-Sparc or Solaris-AMD64. At login, BIRCH determines the OS/hardware platform. Depending on the platform, BIRCH then chooses binaries and libraries appropriate for that system. The BIRCH implementation of GDE can also handle cases in which a program is not available on all platforms. For example, if a program is only available for Solaris-Sparc, the user will see that program in the GDE menus when logged into a Solaris-Sparc host, but not when logged into a Linux-Intel or Solaris-AMD64 host.

In a heterogeneous system, some hosts may have single CPUs and others multiple CPUs. At login BIRCH sets environment variables specifying whether or not threaded applications can take advantage of multiple CPUs.

#### Setting the user environment

Unix/Linux systems achieve a high level of flexibility through use of environment variables, which are used by the shell (command interpreter) to store information such as the locations of programs and files. When a user logs in, BIRCH reads startup scripts that set numerous environment variables for the duration of the session. For example, $BIRCH_PLATFORM tells the OS/hardware platform of the machine to which the user is currently logged in. Since a wide variety of shells are available in Unix/Linux, BIRCH has startup scripts appropriate for most of the major shells (eg. sh, bash, ksn, csh, tcsh). This simplifies things especially for new users, who in most cases don't even know which shell they are using.

Some of the startup scripts are found in $BIRCH/local. Because almost any code could be added to these scripts, the system administrator has great flexibility in tailoring BIRCH to the needs of the system and the user base. For example, the default PDF viewer is set by the statement 'GDE_PDFVIEW=acroread'. On a system that did not have Adobe Reader, the statement could be changed to a different PDF viewer eg. 'GDE_PDFVIEW=ggv'.

First time users run the 'newuser' script to set their accounts to read the BIRCH startup scripts. Consequently, BIRCH does not have to be installed in system directories, but instead, can be administered through a regular user account in a world-readable directory. Therefore, root permissions are not required to install and manage a BIRCH system. Eliminating the need for root access also provides added security.

#### A single view of all documentation and datafiles

**Figure 5:**
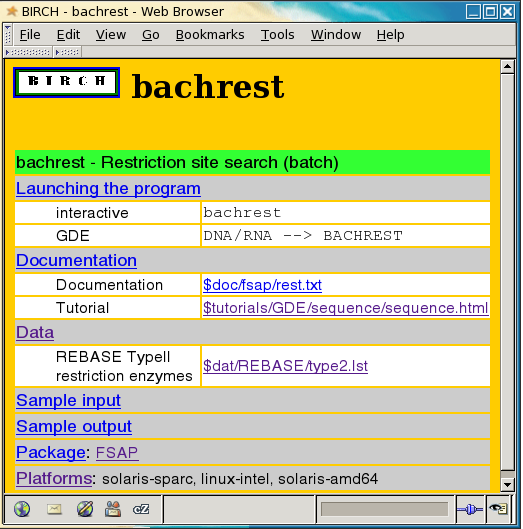
**BIRCH documentation**. Example of a web page for the bachrest restriction site search program, generated from the birchdb database. This entry shows that bachrest can be run as a conversational program in a text window (interactive), or run from the DNA/RNA menu in GDE (GDE). Two documentation files are listed, as well as an ancillary datafile containing a list of restriction enzymes. The Platforms field indicates that bachrest is available on all platforms supported by BIRCH.

One of the problems facing users on a system with many bioinformatics packages is that documentation is often scattered across many locations on the system. The software included with BIRCH is from a wide variety of authors, and documentation is written in different styles (eg. Unix manual pages, tutorials, user's guides), and in many formats (eg. PDF, HTML, text) [13]. To make documentation easy to find, documentation for the core BIRCH programs is catalogued in the birchdb database, and documentation for locally-installed programs is catalogued in the lbirchdb database. Both databases are implemented using ACeDB [26], a small database engine which includes an easy-to-use graphic interface. When BIRCH is installed or updated, the contents of both databases are merged, and a hierarchical set of web documentation pages is generated, including programs listed by category, programs listed by package, and a program index. For each program a separate web page is generated, listing the name and short description of the program, information on how to launch the program, links to documentation and sample datafiles, a listing of os/hardware platforms for which the program is available, as well as a link to the web page describing the package to which the program belongs. An example is seen in Figure 5.

The user doesn't care whether programs are part of the BIRCH core, or are locally-installed. One of the goals of BIRCH is to make the documentation web pages appear as if they were written specifically for the local BIRCH site. This is particularly useful because most first-time BIRCH users will also be using Unix for the first time. Rather than giving the user a generic set of web pages, the BIRCH documentation pages have sections earmarked for system specific information, such as how to log in or which desktops are available. During installation and updating, these sections of the BIRCH web pages are replaced with local content. For example, the email address for the BIRCH administrator at the University of Manitoba is "psgendb@cc.umanitoba.ca". At another site, the email address would be changed in all web pages to that of the local BIRCH administrator eg. "birch@myhost.org".

**Figure 6:**
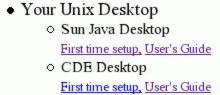
**Replaceable web page components**. This section of the BIRCH home page contains links to documentation for desktops that are installed on the local system. In this example, the BIRCH distribution comes with links to two desktops available on the University of Manitoba Unix system: the Java Desktop and CDE. If BIRCH was installed at a site using the KDE desktop, this section could be replaced with links to KDE documentation. In each case, the 'First time setup' link points to a document which needs to be locally-written at each site, because of differences from site to site. In most cases, all that needs to be done is to modify one of the 'First time setup' documents distributed with BIRCH. The 'User's Guide' link will typically point to a generic User's Guide for a given desktop.

Many sections of the BIRCH web site can be automatically replaced with HTML code specific to the local system. These sections include links to local pages for obtaining and setting up a Unix account, descriptions of local databases, locally-installed software, as well as institutional logos, announcements, and links to local web sites. For example, at the University of Manitoba, both CDE and Sun Java desktops are available. The BIRCH home page contains a set of links to documentation for these desktops as shown in Figure 6. During installation and updating, BIRCH will replace this section with HTML code found in $BIRCH/local.

#### Simplifying BIRCH system administration

BIRCH provides for the system administrator an organizational framework and tools that ensure that programs and documentation remain easily accessible to users. Because startup scripts are read from a central location, the user never needs to perform configuration steps when new software or databases are installed. By the same token, installation and updating of a BIRCH site is automated by birchconfig, the BIRCH install wizard. The BIRCH Administrator's Guide spans numerous topics, including customization of the BIRCH web site, managing systems with multiple servers or operating platforms, installing and merging 3rd party applications into BIRCH, and setting default applications for viewing and displaying data.

BIRCH tries to minimize the skill set needed for being a BIRCH administrator. Where a computer specialist is not available, a biologist with basic knowledge of perhaps 20 of the most common Unix commands, some knowledge of how to write and edit web pages, and some knowledge of shell scripting should be able to install and update a BIRCH system for a lab or working group. Minimizing this skill set has guided the design of BIRCH. Recognizing that tutorials are as important for the system administrator as they are for the user, the BIRCH Administrator's Guide covers all aspects of local customization and addition of new programs with step-by-step instructions, illustrated with screenshots.

## Results and Discussion

One of the problems with the decentralized PC model of computing is that it implicitly makes every user a system administrator. BIRCH frees biologists from system administration tasks, allowing them to focus on being users.

It could be argued that the single biggest limiting factor in bioinformatics is not hardware or software or algorithms, but the high learning curve needed to work with all but the simpler tools. By handling the minor technical details, the learning curve is shortened, allowing the user to concentrate on the theoretical considerations of running the program. For tasks such as phylogenetic analysis, in which numerous steps are required, analysis pipelines can be built into a single menu. At the same time, inclusion of programs in a pipeline does not negate the ability to use each of them as standalone programs, where finer control is needed for intermediate steps.

While GDE is not itself an object-oriented application, the BIRCH implementation of GDE uses some OO strategies, within the limitations imposed by the diverse ways in which different programs represent the same types of data. Most importantly, four GDE interfaces allow data to have some of the behavior of objects. For example, a phylogenetic tree, regardless of how it was generated, will appear in a tGDE window. The 'methods' of a tree object would be implemented in the menus that appear in tGDE. At a lower level of implementation, where there are commonalities in data types, a single script or program often handles input or output for many programs. For example, a single program parses the IDs for hits from BLAST or FASTA, and sends output to files, viewers, or even dGDE. In the latter case, the IDs are in the form of a list, which could be used as input for databases queries.

BIRCH is flexible enough to accommodate programs written any language, and documentation in many different formats. While it is straightforward to install many software packages side by side on any computer system, there are usually few mechanisms for integrating them into a single view for the user. In principle, programs and documentation could be added to a local copy of any bioinformatics package. Depending on the package, it might be necessary to revise programs and documentation to be added to adhere to a specific requirements, such as an API, or documentation format, respectively. These changes would have to be re-integrated each time the package was updated. BIRCH follows the opposite strategy. Since integration of local add-ons is a standard part of a BIRCH update, updates do not break local changes.

As described above, BIRCH is also unique in supporting a heterogeneous computing environment with multiple os/hardware platforms coexisting. To make this possible, there is only one version of BIRCH, not one for each platform. BIRCH is organized around a core of platform-neutral scripts and Java programs. Platform-specific binaries and libraries are downloaded separately. While BIRCH currently supports three platforms (Solaris-Sparc, Solaris-AMD64, and Linux-Intel) it would be straightforward to support other platforms. In fact, adapting BIRCH from Solaris-Sparc to Solaris-AMD64 required less than one person/week.

## Conclusion

BIRCH is designed from the perspective of the biologist, keeping in mind that ease of use is highly dependent on ease of system management. For labs or institutions interested in setting up a central bioinformatics system, BIRCH is a quick way of getting started. At the same time, the organization and administration of an existing collection of programs could be improved by integrating these into a local copy of BIRCH.

## Availability and requirements

Project name: BIRCH

Project home page: 

Operating systems: Solaris, Linux

Other requirements: sh, csh, Python, Java

Restrictions: none
